# Aphids (Hemiptera: Aphididae) in Living Collections of Selected European Botanic Gardens: Diversity, Biosecurity Challenges, and Sentinel Insights

**DOI:** 10.3390/insects17020196

**Published:** 2026-02-12

**Authors:** Karina Wieczorek, Dominik Chłond, Kaja Ball, Agnieszka Zawisza-Raszka, Kenneth Bauters, Dirk Baert, Matt Elliot

**Affiliations:** 1 Institute of Biology, Biotechnology and Environmental Protection, Faculty of Natural Sciences, University of Silesia in Katowice, 40-007 Katowice, Poland; 2 Faculty of Biology, Jagiellonian University, 30-387 Kraków, Poland; 3 Municipal Botanic Garden in Zabrze, 41-800 Zabrze, Poland; 4 Botanic Garden en Herbarium at the Ghent University, 9000 Gent, Belgium; 5 WAKONA Natuurstudiewerkgroep Waasland-Noord, 9100 Sint-Niklaas, Belgium; 6 Royal Botanic Garden Edinburgh, Edinburgh EH3 5NZ, UK

**Keywords:** alien species, expert bioblitz, host plant, insect, invasion, monitoring, pest

## Abstract

Botanic gardens host diverse living plant collections and are increasingly recognized as sentinel sites for documenting insect biodiversity and detecting biological invasions. Aphids (Hemiptera: Aphididae) are well suited to such monitoring due to their close host associations, rapid population growth, and importance as horticultural pests and virus vectors. Here, we document the aphid fauna recorded in five European botanic gardens—Zabrze (Poland), Meise (Belgium), and the Royal Botanic Gardens in Edinburgh, Benmore, and Logan (UK)—based on surveys conducted in 2022 and 2023. Sampling approaches included short-duration expert bioblitz-style surveys and extended seasonal monitoring. In total, more than one hundred aphid species were recorded across all sites. Observed species lists differed among gardens and survey periods, reflecting variation in sampling timing, intensity, and host plant composition. Several alien aphid species to Europe of Oriental and Nearctic origin were detected, including multiple new national records. Alien taxa were found both in outdoor living collections and in controlled environments such as glasshouses, nurseries, restricted areas, and plant shops, which may function as entry points as well as locations for early intervention. These findings illustrate the value of botanic gardens for documenting aphid diversity and supporting early detection of non-native species relevant to plant health and biosecurity.

## Simple Summary

Botanic gardens host diverse living plant collections that attract a wide range of insects, including aphids, some of which may become invasive pests. This study documents aphid species recorded in selected European botanic gardens and highlights the role of these institutions as early-warning sites for alien aphids relevant to plant health and biosecurity.

## 1. Introduction

Botanic gardens represent unique and increasingly important sites for biodiversity conservation, horticultural development, and ecological research. Globally, about 4500 botanic gardens maintain living collections encompassing over 100,000 plant taxa [1]. As curated living collections, they host a wide diversity of native and non-native plants, often growing in proximity under highly managed conditions. This mosaic of hosts creates novel habitats not only for plants but also for a range of associated insect species, including herbivores, pollinators, predators, and potential pests. In addition to their horticultural and educational functions, botanic gardens are increasingly recognized as sentinel systems for the early detection of invasive species and as monitoring platforms for biotic interactions in urbanized and semi-natural landscapes, a role formalized through their incorporation into the International Plant Sentinel Network (IPSN) [2,3,4]. Their fixed location, long-term management history, and documented plant inventories make them particularly suitable for standardized biodiversity assessments and for evaluating the ecological consequences of human-mediated plant movements [5,6,7].

Among insects, aphids (Hemiptera: Aphididae) are especially well-suited to garden-based monitoring. With more than 5000 described species worldwide [8], they represent one of the most ecologically and economically significant groups of plant-feeding insects [9]. Aphids exhibit high host specificity, short generation times, and frequent parthenogenetic reproduction, making their populations sensitive to shifts in host availability, microclimatic conditions, and horticultural management. Several species are major agricultural or horticultural pests, not only through direct feeding but also as vectors of plant viruses [10,11]. Others are known invaders: Europe has seen a steady increase in alien aphid species, often introduced through the international trade of ornamental plants [12,13,14]. These characteristics underscore their dual ecological and economic importance, and support their use as species indicators in managed plant systems.

Botanic gardens have long been recognized as valuable sites for faunistic surveys of aphids due to their high host plant diversity; however, most existing studies are restricted to single gardens or short observation periods and were not explicitly designed to support early-warning or biosecurity objectives [15,16,17,18,19,20,21,22,23,24,25]. In recent years, rapid-assessment formats such as bioblitz-style surveys have gained attention as efficient tools for short, high-intensity inventories in managed landscapes, including botanic gardens [26]. When conducted by trained specialists, expert-led bioblitz surveys can generate high-quality faunistic data within limited time frames, particularly for insects such as aphids whose detection depends strongly on season, host condition, and concentrated search effort [27]. In botanic gardens, such surveys can complement longer seasonal observations by enabling rapid coverage of taxonomically diverse living collections.

A fundamental feature of aphid biology is their close association with host plants, often restricted to one or a few closely related taxa [9]. Because plants cultivated in botanic gardens are identified and curated, these institutions provide well-documented settings for recording aphid–host associations and detecting non-native species. Aphid assemblages recorded in botanic gardens are also shaped by multiple, interacting factors. Garden size and plant richness influence aphid occurrence primarily through the availability of suitable host plants, while the cultivation of non-native taxa increases the likelihood of encountering alien or newly arrived aphid species. Climatic conditions such as temperature and rainfall further affect aphid activity, colony persistence, and detectability, and survey timing determines which life stages and host associations are observed. Consequently, the composition of aphid assemblages documented in gardens reflects both ecological and the temporal context of sampling.

By combining expert bioblitz-style intensive surveys with extended seasonal sampling across five botanic gardens in Poland, Belgium, and the United Kingdom, this study reports results of a coordinated multi-garden, qualitative survey documenting aphid diversity and alien species in living collections.

## 2. Materials and Methods

### 2.1. Study Sites

**Figure 1:**
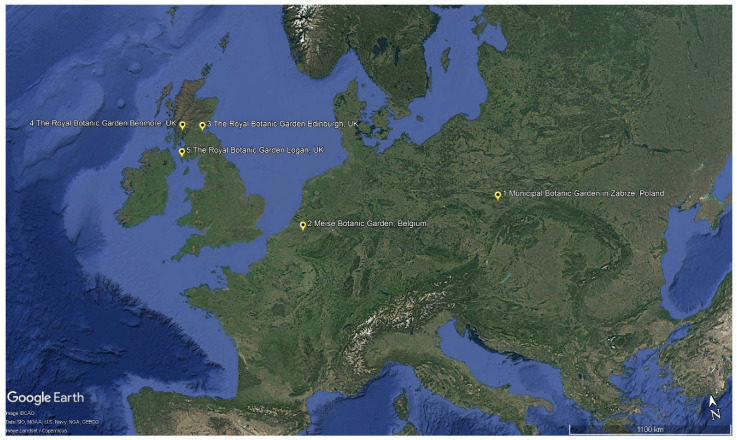
Locations of the five botanic gardens surveyed in this study: (1) Municipal Botanic Garden in Zabrze (Poland), (2) Meise Botanic Garden (Belgium), (3) The Royal Botanic Garden Edinburgh (UK), (4) The Royal Botanic Garden Benmore (UK), (5) The Royal Botanic Garden Logan (UK).

Aphid surveys were carried out in five European botanic gardens representing various geographic locations (Figure 1), contrasting sizes, climates, and living collections. Basic descriptors of each garden are given below.

#### 2.1.1. Municipal Botanic Garden in Zabrze, Poland (MBG Zabrze)—50°17′44.27″ N, 18°45′51.46″ E

**Figure 2:**
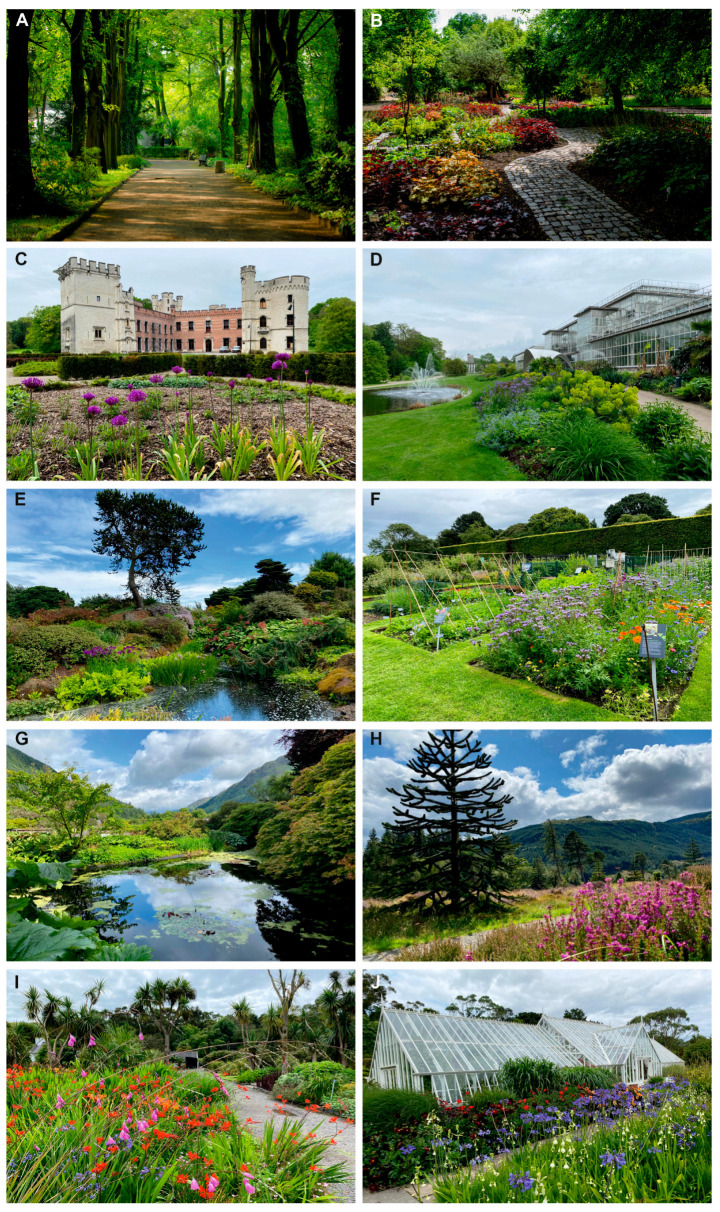
(**A**) MBG Zabrze, Poland—Maple Path; (**B**) MBG Zabrze—National Collection of *Heuchera*; (**C**) Meise BG, Belgium—Bouchout Castle; (**D**) Meise BG—Orangery; (**E**) RBG Edinburgh, UK—Rock Garden; (**F**) RBG Edinburgh—Vegetable Garden; (**G**) RBG Benmore, UK—Pond; (**H**) RBG Benmore—The Chilean Rainforest; (**I**) RBG Logan, UK—South Woodland; (**J**) RBG Logan—Conservatory. Photo (**A**,**B**): A. Zawisza—Raszka; Photo (**C**–**J**): K. Wieczorek.

This relatively small municipal garden (6.36 ha), located in southern part of Poland, maintains a collection of 1500 plant taxa. The living collections include both native wild Central European flora and ornamental exotic species arranged in themed outdoor beds and greenhouses (Figure 2A,B). The regional climate is temperate continental, with cold winters and warm summers [28].

#### 2.1.2. Meise Botanic Garden, Belgium (Meise BG)—50°55′37.12″ N, 4°20′6.59″ E

This is one of the largest botanic gardens in Europe (92 ha), with a living collection of 18,000 plant taxa, including extensive temperate and subtropical assemblages. The garden also maintains several large greenhouses housing tropical flora (Figure 2C,D). The climate is temperate oceanic with relatively mild winters [29].

#### 2.1.3. The Royal Botanic Garden Edinburgh, UK (RBG Edinburgh)—55°57′54.04″ N, 3°12′32.77″ W

This is the main garden of the Royal Botanic Garden Edinburgh network, covering 28.33 ha and a living collection of 14,781 plant taxa, with a strong focus on temperate floras of the Northern Hemisphere (Figure 2E,F). The climate is cool temperate oceanic, characterized by mild summers and relatively high rainfall [30].

#### 2.1.4. The Royal Botanic Garden Benmore, UK (RBG Benmore)—56°1′34.26″ N, 4°58′51.08″ W

This is a large regional garden (48.56 ha) situated in a mountainous valley in western Scotland hosting 2432 plant taxa. It specializes in conifers and extensive rhododendron collections (Figure 2G,H). The mild oceanic climate, influenced by high rainfall, supports luxuriant growth of Himalayan and Pacific Northwest taxa [31].

#### 2.1.5. The Royal Botanic Garden Logan, UK (RBG Logan)—54°44′35.49″ N, 4°57′31.86″ W

A coastal garden (6.1 ha, 2378 plant taxa) located in southwest Scotland, benefiting from a particularly mild microclimate due to the influence of the Gulf Stream. This permits the successful cultivation of subtropical and Mediterranean plant taxa rarely grown outdoors in Britain (Figure 2I,J) [32].

### 2.2. Timing and Sampling Procedure

Aphid surveys were conducted during the following periods: Municipal Botanic Garden in Zabrze (Poland): 5 April–5 October 2023; Meise Botanic Garden (Belgium): 21–22 May 2022 and 1–10 May 2023; Royal Botanic Garden Edinburgh (UK): 5–12 July 2023; Royal Botanic Garden Benmore (UK): 17–20 July 2023; Royal Botanic Garden Logan (UK): 24–25 July 2023. In the Meise Botanic Garden (21–22 May 2022) and Zabrze Botanic Garden (29 May 2023), we additionally conducted expert bioblitz-style surveys. These were short-duration, high-intensity surveys performed exclusively by trained specialists, designed to maximize the proportion of the living collections inspected within a limited time frame.

Surveys were conducted using a combination of visual inspection of host plants and direct collection of aphids. During each visit, the collector applied a standardized qualitative visual-search protocol. All major sections of each garden, including arboreta, themed outdoor beds, nurseries, and, where accessible, glasshouses, were walked systematically along the main path network. Rather than sampling a fixed number of plants, all dominant woody hosts and a broad representation of herbaceous and ornamental species were examined, ensuring wide coverage of the living collections. Plants were inspected using an expert-guided host-oriented search strategy. During systematic walking through each garden section, attention was directed primarily toward plant taxa known to host aphids, based on established aphid–host associations (e.g., cultivated ornamentals, common woody genera, and managed collections). Within these hosts, plant parts most likely to harbor aphids were examined, regardless of whether colonies or single individuals were immediately visible. Young shoots, both surfaces of leaves, stems, petioles, terminal buds, and inflorescences were checked, i.e., the plants’ parts where aphid colonies typically develop; heavy infestation refers here to conspicuous, dense colonies readily detectable during visual inspection. In accordance with horticultural and conservation restrictions in botanic gardens, sampling was strictly non-destructive, and no above- or below-ground plant parts were intentionally disturbed during aphid collection. In each garden, each field visit focused on a different section of the living collections. Additional opportunistic checks were made on plants located outside formally designated collection areas (e.g., arboreta or themed beds), on plants near buildings, service areas, and unguided paths. This continuous-search approach maximized host coverage while remaining compatible with the conservation and display requirements of botanic gardens. The present survey was an aphid-focused faunistic and sentinel investigation; therefore, records of host plants without aphids were not collected.

The aphids were collected directly from the host plant using a fine brush and transferred into Eppendorf tubes containing 70% ethanol for subsequent identification and analysis.

### 2.3. Species Identification

Adult wingless (apterous viviparous) and winged (alate viviparous) females were slide-mounted using the method of Wieczorek [33] and examined with a Nikon Ni-U light microscope equipped with a phase contrast system and DS-Fi2 camera Nikon Corporation (Tokyo, Japan). Species determination was based primarily on morphological characters, following standard identification keys [9,34,35,36]. Nomenclature was standardized using [8]. Alien species and new country records were flagged according to published distributional data and national aphid checklists [37,38,39]. Voucher specimens were deposited in the entomological collection of the University of Silesia in Katowice, Poland (DZUS).

Samples collected during the expert bioblitz survey in Meise Botanic Garden (May 2022) were not retained as physical specimens; these records were confirmed by image-based identification from high-resolution photographs. While photographic material may not allow full verification of all characters, identifications were restricted to taxa showing unambiguous diagnostic features, ensuring reliability of these records.

Detailed collection data for all studied species including sample number, date of collection, locality, host plant, aphid species name, voucher number and name of collector are provided in Tables S1–S6.

The recommended Practical protocol for monitoring aphids in botanic gardens presented in Table S7 synthesizes the core methodological elements applied in this study and formalizes them into a recommended framework for future surveys; not all elements were implemented uniformly at every site due to logistical and access constraints.

The source for the botanical nomenclature was the International Plant Names Index [40].

The outline Europe map was obtained from “https://earth.google.com (accessed on 2 September 2025)”.

The figures were prepared using Corel Draw ver. 23 (Corel Corporation, Ottawa, ON, Canada).

## 3. Results

The list of aphid species recorded in all studied botanic gardens, with alien species and new country records flagged, is presented in Table 1, with full taxonomic details including higher classification and species authorities.

### 3.1. Municipal Botanic Garden in Zabrze

A total of 152 aphid samples were collected during ten visits between April and October 2023. In total, 60 species of Aphididae belonging to 32 genera and nine subfamilies were identified. The subfamily Aphidinae was the most species-rich, represented by 19 genera and 41 species. The genus *Aphis* Linnaeus was the most diverse, with 14 species. The remaining genera were represented by one to seven species each. Twelve species (20% of the MBG Zabrze aphid fauna) were regarded as alien to the European aphid fauna, including six species of Oriental origin, three of Nearctic origin, two of Tropical origin, and one cryptogenic. The identified host plants included 92 species from 79 genera. The genus *Acer* was most frequently infested, with eight taxa hosting five aphid species. Most aphids recorded were monophagous or narrow oligophages. Among polyphagous taxa, *Macrosiphum* (*M.*) *euphorbiae* and *Aphis* (*A.*) *fabae fabae* were the most widespread, occurring on 12 and ten host plant taxa, respectively. Aphids were collected from both ornamental plants, including exotic species and spontaneous native vegetation, often showing heavy infestations. They were found throughout the garden, with the greatest species variety recorded on herbaceous plant beds during the survey. Two species—*Myzus* (*N.*) *persicae* and *M.* (*S.*) *ascalonicus*—were found exclusively on ornamental plants cultivated in glasshouses. Although aphids were collected throughout the entire growing season, a single intensive field visit on 29 May 2023, conducted by two collectors, yielded a total of 41 aphid species belonging to 27 genera and six subfamilies. Within this dataset, *Takecallis arundinariae* represent new country records for Poland [41], while *Paczoskia longipes* is an uncommon species, rarely collected in Central Europe.

### 3.2. Meise Botanic Garden

A total of 72 aphid samples were collected during seven visits in early May 2023. In total, 31 species of Aphididae belonging to 17 genera and six subfamilies were identified. The subfamily Aphidinae was the most species-rich, represented by ten genera and 18 species. A similar species share was found within individual genera, from single to a maximum of four species. The most frequently collected aphids were from the genus *Periphyllus*—17 samples, associated with various *Acer* host plants. Nine species (29% of the Meise BG aphid fauna) were regarded as alien to the European aphid fauna, including five species of Oriental origin, three of Nearctic origin and one of Tropical origin. The identified host plants included 61 species from 36 genera. The genus *Acer* was most frequently infested, with 12 taxa hosting four aphid species. Most aphids recorded were monophagous or narrow oligophages. Among polyphagous taxa, *Macrosiphum* (*M.*) *euphorbiae* was the most widespread, occurring on seven host plant taxa. Aphids were collected mostly from ornamental plants, including exotic species. However, the main sources of Aphidinae taxa were the Culinary Garden and the Scent and Colours Garden, where heavy infestations were observed on ornamental and cultivated plants. Similarly, heavy infestation of *Periphyllus californiensis* was found exclusively on various varieties of *Acer palmatum*, presented in a garden shop.

In addition, a similar survey was conducted during a two-day visit on 21–22 May 2022, when 51 aphid species belonging to 27 genera and six subfamilies were identified. The subfamily Aphidinae was the most species-rich, represented by 14 genera and 31 species. The genus *Aphis* was the most diverse, with eight species. Eleven species (21% of the aphid fauna) were regarded as alien to the European aphid fauna, including seven species of Oriental origin, two of Nearctic origin, one of Tropical origin, and one cryptogenic. The identified host plants included 35 species from 42 genera.

Comparing both surveys, 13 aphid species were recorded in common, while differences in species composition largely reflected the different timing of sampling, with the 2022 survey taking place in late May and the 2023 survey covering the early spring season. In total, over two research seasons conducted in spring (May inclusive), 67 species of aphids were collected, including 17 species of foreign origin. Within this dataset, 12 species appear to represent new country records for Belgium.

### 3.3. The Royal Botanic Garden Edinburgh

A total of 66 aphid samples were collected during six visits in July 2023. In total, 32 species of Aphididae belonging to 23 genera and six subfamilies were identified. Two subfamilies were characterized by the greatest species diversity—Calaphidinae with ten genera and 11 species, and Aphidinae with nine genera and 14 species. Nine species (28% of the RBG Edinburgh aphid fauna) were regarded as alien to the European aphid fauna, including five species of Oriental origin, three of Nearctic origin and one of Tropical origin. The identified host plants included 54 species from 37 genera. The genus *Betula* was most frequently infested, with seven taxa hosting five aphid species. The highest taxonomic diversity of Aphidinae was detected in the areas surrounding the Botanic Cottage and in the Vegetable Garden. Most aphids recorded were monophagous or narrow oligophages; among polyphagous taxa, *Macrosiphum* (*M.*) *euphorbiae* was the most widespread, occurring on seven host plant taxa. Collected aphids were primarily associated with tree hosts; smaller amount was detected from ornamental plants, including exotic species (e.g., the critically endangered South African endemic *Gladiolus cruentus* infested by *Myzus* (*M.*) *ornatus*). Heavy infestations of species belonging to the genera *Aphis*, *Cavariella* Del Guercio and *Pterocomma* Buckton were also found on *Salix* species maintained in the restricted area of nursery.

### 3.4. The Royal Botanic Garden Benmore

A total of 20 aphid samples were collected during three visits in July 2023. In total, 11 species of Aphididae belonging to 11 genera and four subfamilies were identified. The subfamily Calaphidinae was the most species-rich, with seven species each representing a single genus. One species (9% of the RBG Benmore aphid fauna), *Takecallis nigroantennatus* associated with *Yushania anceps*, a bamboo native to the central and north-western Himalayas, was regarded as alien to the European aphid fauna. The identified host plants included 16 species from ten genera. The genus *Betula* was most frequently infested, with four taxa hosting two aphid species. Most aphids recorded were monophagous or narrow oligophages, associated with tree hosts.

### 3.5. The Royal Botanic Garden Logan

A total of 11 aphid samples were collected during two visits in July 2023. In total, six species of Aphididae belonging to four genera and two subfamilies were identified. Three species (50% of the RBG Logan aphid fauna) were regarded as alien to the European aphid fauna, including two of Oriental origin and one of Nearctic origin. The identified host plants included eight species from eight genera. *Macrosiphum* (*M.*) *euphorbiae* was the most widespread polyphagous species, occurring on five host plant taxa, with heavy infestations detected on plants in the Logan Conservatory and the isolation glasshouse of restricted area.

## 4. Discussion

This study provides an overview of aphid assemblages recorded in living plant collections across selected European botanic gardens. The results document aphid occurrence, host associations, and alien species detection that are directly relevant to the role of botanic gardens as sentinel sites for plant health surveillance. By integrating expert-led bioblitz-style surveys with targeted seasonal sampling across multiple gardens, this work establishes a baseline inventory of aphid assemblages that can support future monitoring and the early detection of alien species.

Across all gardens, aphid assemblages included both widespread native taxa and alien species associated with cultivated plants. Several rarely recorded or newly detected species were found, confirming that botanic gardens can act as effective observation points for aphid diversity, including taxa that may be overlooked in conventional agricultural or natural habitats. In urban and managed landscapes, such gardens therefore also function as refugia for native aphids, while simultaneously serving as early-warning locations for new introductions [14,19,22,24,42].

A key observation from this study is the effectiveness of short, expert bioblitz-style surveys. In both Meise (2022) and Zabrze (2023), high-intensity sampling conducted by trained aphid specialists during a limited time window yielded a substantial proportion of the total aphid diversity recorded. Although such surveys do not replace full-season monitoring, they proved efficient for detecting a broad range of species active during peak periods. These findings support the use of expert-led rapid surveys as a practical and scalable tool for aphid monitoring in botanic gardens, particularly when time or resources are limited.

Host plant associations were central to the aphid assemblages observed. Aphids were most frequently recorded on woody plants, ornamental collections, and cultivated taxa known to host diverse aphid faunas. Several notable associations highlight the ecological and conservation relevance of botanic gardens. For example, infestations of *Myzus* (*M.*) *ornatus* on *Gladiolus cruentus*, a critically endangered South African endemic species, illustrate how curated collections can act as contact zones where specialized herbivores exploit threatened ex situ hosts. Similarly, records of *Macrosiphum* (*M.*) *euphorbiae* on *Heuchera* spp. within a National Collection deposited in the MBG Zabrze, Poland demonstrate how living collections can support both common generalists and host-associated species. Conversely, some gardens with highly diverse but biogeographically atypical plant assemblages supported relatively few aphid species outdoors. In Logan, most aphids were detected under controlled conditions in nurseries or conservatories, while outdoor displays dominated by Australasian, South American, or African flora yielded few records. This pattern likely reflects a mismatch between host plant origin and the native distribution of Aphididae, combined with seasonal effects, rather than an absence of suitable sampling effort.

Alien aphids were documented in all surveyed gardens, underscoring the biosecurity relevance of living collections. Restricted areas such as nurseries, isolation glasshouses, and plant shops emerged as particularly important points for both introduction and control. Several detections illustrate this role clearly. In Meise, infestations of *Periphyllus californiensis* on imported *Acer palmatum* were detected rapidly and eradicated by garden staff (personal communication with garden staff). Outdoor collections also functioned as long-term reservoirs for non-native aphids, such as *Eriosoma lanigerum* on old apple varieties. In the United Kingdom, records of *Cinara curvipes*, *Ericolophium holsti*, and *Essigella californica* [24,43] extended known distribution ranges northwards, demonstrating how botanic gardens can provide the first national or regional records of alien aphids. These observations emphasize that botanic gardens and arboreta operate as frontline sentinel sites, not only for tracking the spread of established invaders but also for detecting new or emerging species at an early stage. The presence of alien aphids across multiple garden types further highlights the importance of targeted surveillance in high-risk zones associated with plant movement and trade.

The results of this study reinforce the role of botanic gardens as key components of international sentinel networks for invasive pest monitoring [44,45]. Both Meise Botanic Garden and the Royal Botanic Garden Edinburgh are members of the International Plant Sentinel Network, which coordinates global surveillance of emerging threats through living plant collections. Our findings demonstrate that aphids, including alien taxa, are readily detectable in curated collections and may be identified at an early stage of establishment, underscoring the value of botanic gardens as early-warning sites for pests that are rare, overlooked, or not yet recognized as harmful in their native ranges. Such expert-led bioblitz-style surveys can be readily implemented through targeted collaboration between botanic garden staff and aphid specialists, providing an efficient tool for routine monitoring without requiring formal citizen–science frameworks.

Beyond early detection, botanic gardens can actively reduce the risk of pest spread through targeted management actions. Routine inspection of incoming plant material, short quarantine periods for new accessions, and focused monitoring of high-risk areas such as nurseries, glasshouses, and plant shops can help interrupt potential invasion pathways. Integrating such practices into garden management, together with standardized monitoring protocols, transparent data sharing, and collaboration with national plant protection organizations, would further strengthen the role of botanic gardens as a frontline resource for plant health surveillance and global biosecurity [46,47].

## 5. Conclusions

Botanic gardens represent priority sites for aphid monitoring, as their diverse and well-documented living collections enable the early detection of alien and horticulturally significant species. This study shows that expert-led qualitative surveys, including short, bioblitz-style sampling, can efficiently document aphid assemblages under real-world management conditions and provide a practical baseline for future monitoring. Integrating such surveys into routine garden management, particularly in high-risk areas such as nurseries, glasshouses, and plant exchange facilities, would strengthen early detection and rapid response efforts and enhance the contribution of botanic gardens to plant health and biosecurity frameworks.

## Figures and Tables

**Table 1:** List of aphids (Aphididae) collected in: the Municipal Botanic Garden in Zabrze, Poland (2023); Meise Botanic Garden, Belgium (2022, 2023); the Royal Botanic Gardens in Edinburgh, Benmore and Logan, UK (2023). ! (marked lines)—species alien to Europe; *—new country records.

No.	Taxon	MBG Zabrze Poland	Meise BG Belgium	Meise BG Belgium	RBG Edinburgh UK	RBG Benmore UK	RBG Logan UK
		2023	2022	2023	2023	2023	2023
Eriosomatinae							
1	! *Eriosoma lanigerum* (Hausmann, 1802)	+		+			
2	*Prociphilus* (*Stagona*) *xylostei xylostei* (De Geer, 1773)	+					
Anoeciinae							
3	*Anoecia* (*Anoecia*) *corni* (Fabricius, 1775)	+		+			
Mindarinae							
4	*Mindarus abietinus* Koch, 1857	+	+				
Drepanosiphinae							
5	*Drepanosiphum acerinum* (Walker, 1848)	+					
6	*Drepanosiphum platanoidis* (Schrank, 1801)	+	+		+	+	
7	*Drepanosiphum oregonense* Granovsky, 1939				+		
Phyllaphidinae							
8	*Phyllaphis fagi* Linnaeus, 1767		+	+	+	+	
Calaphidinae: Calaphidini							
9	*Betulaphis quadrituberculata* (Kaltenbach, 1843)				+		
10	*Calaphis flava* Mordvilko, 1928				+	+	
11	*Euceraphis betulae* (Koch, 1855)	+			+	+	
12	*Euceraphis punctipennis* (Zetterstedt, 1828)		+		+		
13	*Monaphis antennata* (Kaltenbach, 1843)				+		
Calaphidinae: Panaphidini							
14	*Eucallipterus tiliae* (Linnaeus, 1758)	+	+		+	+	
15	*Agrioaphis castanicola* Baker, 1917	+	+	+	+		
16	*Myzocallis* (*Myzocallis*) *carpini* (Koch, 1855)	+	+				
17	*Myzocallis* (*Myzocallis*) *coryli* (Goeze, 1778)	+	+	+	+	+	
18	! *Panaphis juglandis* (Goeze, 1778)			+			
19	*Pterocallis* (*Pterocallis*) *alni* (De Geer, 1773)		+			+	
20	! *Takecallis arundicolens* (Clarke, 1903)		+ *	+ *			+
21	! *Takecallis arundinariae* (Essig, 1917)	+ *	+ *	+ *	+		+
22	! *Takecallis nigroantennatus* Wieczorek, 2023		+ *	+ *	+	+	
23	! *Takecallis taiwanus* (Takahashi, 1926)		+ *				
24	*Tinocallis* (*Sappocallis*) *nevskyi* Remaudière, Quednau & Heie, 1988		+		+		
25	! *Tinocallis* (*Sappocallis*) *takachihoensis* Higuchi, 1972		+ *				
26	*Tuberculatus* (*Tuberculoides*) *annulatus* (Hartig, 1841)	+	+				+
27	*Tuberculatus* (*Tuberculoides*) *neglectus* (Krzywiec, 1966)				+	+	
Chaitophorinae: Chaitophorini							
28	*Chaitophorus capreae* (Mosley, 1841)		+				
29	*Chaitophorus populeti populeti* (Panzer, 1804)	+					
30	*Periphyllus acericola* (Walker, 1848)	+	+	+			
31	*Periphyllus aceris* (Linnaeus, 1776)	+			+		
32	*Periphyllus coracinus* (Koch, 1854)			+			
33	! *Periphyllus californiensis californiensis* (Shinji, 1917)	+		+ *			
34	*Periphyllus testudinaceus* (Fernie, 1852)	+		+			
Aphidinae: Aphidini							
35	*Aphis* (*Aphis*) *armata* Hausmann, 1802					+	
36	*Aphis* (*Aphis*) *craccivora* Koch, 1854	+					
37	*Aphis* (*Aphis*) *fabae fabae* Scopoli, 1763	+	+	+	+		+
38	*Aphis* (*Aphis*) *farinosa farinosa* Gmelin, 1790				+		
39	! *Aphis* (*Aphis*) *gossypii gossypii* Glover, 1877	+	+				
40	*Aphis* (*Aphis*) *hederae* Kaltenbach, 1843	+					
41	*Aphis* (*Aphis*) *ilicis* Kaltenbach, 1843		+				
42	*Aphis* (*Aphis*) *nerii* Boyer de Fonscolombe, 1841	+					
43	*Aphis* (*Aphis*) *newtoni* Theobald, 1927	+					
44	*Aphis* (*Aphis*) *rumicis* Linnaeus, 1758	+	+	+			
45	*Aphis* (*Aphis*) *sambuci* Linnaeus, 1758	+	+				
46	*Aphis* (*Aphis*) *sedi* Kaltenbach, 1843	+			+		
47.	*Aphis* (*Aphis*) *solanella* Theobald, 1914	+					
48	! *Aphis* (*Aphis*) *spiraecola* Patch, 1914	+	+ *		+		
49	! *Aphis* (*Aphis*) *spiraephaga* Müller, 1961	+					
50	*Aphis* (*Aphis*) *taraxacicola* (Börner, 1940)		+				
51	*Aphis* (*Aphis*) *urticata* Gmelin, 1790	+	+				
52	*Aphis* (*Aphis*) *viburni* Scopoli, 1763	+			+		
53	! *Rhopalosiphum maidis* (Fitch, 1856)	+					
54	*Rhopalosiphum nymphaeae* (Linnaeus, 1761)		+				
Aphidinae: Macrosiphini							
55	*Acyrthosiphon* (*Acyrthosiphon*) *pisum pisum* Harris, 1776				+		
56	*Amphorophora* (*Amphorophora*) *gei* (Börner, 1939)	+					
57	*Amphorophora* (*Amphorophora*) *rubi* (Kaltenbach, 1843)		+	+			
58	*Aulacorthum* (*Aulacorthum*) *langei* Börner, 1939	+					
59	*Aulacorthum* (*Aulacorthum*) *solani solani* (Kaltenbach, 1843)	+	+				
60	*Brachycaudus* (*Acaudus*) *klugkisti* (Börner, 1942)			+			
61	*Brachycaudus* (*Appelia*) *tragopogonis* (Kaltenbach, 1843)			+			
62	*Brachycaudus* (*Brachycaudus*) *helichrysi* (Kaltenbach, 1843)	+					
63	*Brachycaudus* (*Prunaphis*) *cardui* (Linnaeus, 1758)		+	+			
64	*Brevicoryne brassicae* (Linnaeus, 1758)			+			
65	*Capitophorus elaeagni* (Del Guercio, 1894)	+					
66	*Cavariella* (*Cavariella*) *aegopodii* (Scopoli, 1763)				+		
67	*Cavariella* (*Cavariella*) *archangelicae* (Scopoli, 1763)		+				
68	*Cavariella* (*Cavariella*) *theobaldi* (Gillete & Bragg, 1918)		+				
69	*Corylobium avellanae* (Schrank, 1801)	+					
70	*Cryptomyzus* (*Cryptomyzus*) *galeopsidis* (Kaltenbach, 1843)		+	+			
71	*Cryptomyzus* (*Cryptomyzus*) *ribis* (Linnaeus, 1758)		+				
72	*Dysaphis* (*Pomaphis*) *plantaginea* (Passerini, 1860)	+					
73	! *Ericolophium holsti* (Takahashi, 1935)				+		
74	*Hyperomyzus* (*Hyperomyzus*) *lactucae* (Linnaeus, 1758)		+				
75	! *Illinoia* (*Illinoia*) *liriodendri* (Monell, 1879)		+ *				
76	! *Illinoia* (*Masonaphis*) *lambersi* (MacGillivray, 1960)	+					
77	*Liosomaphis berberidis* (Kaltenbach, 1843)	+		+	+	+	
78	*Macrosiphoniella* (*Macrosiphoniella*) *artemisiae artemisiae* (Boyer de Fonscolombe, 1841)		+				
79	*Macrosiphoniella* (*Macrosiphoniella*) *millefolii* (De Geer, 1773)		+		+		
80	*Macrosiphoniella* (*Macrosiphoniella*) *tanacetaria* (Kaltenbach, 1843)	+	+				
81	*Macrosiphoniella* (*Phalangomyzus*) *oblonga* (Mordvilko, 1901)		+				
82	*Macrosiphoniella* (*Phalangomyzus*) *persequens* (Walker, 1852)	+					
83	*Macrosiphum* (*Macrosiphum*) *cholodkovskyi* (Mordvilko, 1909)	+					
84	! *Macrosiphum* (*Macrosiphum*) *euphorbiae* (Thomas, 1878)	+	+	+	+		+
85	*Macrosiphum* (*Macrosiphum*) *euphorbiellum* Theobald, 1917		+ *				
86	*Macrosiphum* (*Macrosiphum*) *gei* (Koch, 1855)	+	+				
87	*Macrosiphum* (*Macrosiphum*) *hellebori* Theobald &Walton, 1923		+	+			
88	*Macrosiphum* (*Macrosiphum*) *knautiae* Holman, 1972			+ *			
89	*Macrosiphum* (*Macrosiphum*) *rosae rosae* (Linnaeus, 1758)	+	+	+	+		+
90	*Megoura viciae* Buckton, 1876		+				
91	*Metopeurum fuscoviride fuscoviride* Stroyan, 1950	+					
92	*Metopolophium* (*Metopolophium*) *dirhodum* (Walker, 1849)		+				
93	! *Myzus* (*Myzus*) *ornatus* Laing, 1932	+			+		
94	*Myzus* (*Nectarosiphon*) *myosotidis* (Börner, 1950)	+					
95	! *Myzus* (*Nectarosiphon*) *persicae* (Sulzer, 1776)	+	+				
96	! *Myzus* (*Sciamyzus*) *ascalonicus* Doncaster, 1946	+	+				
97	*Nasonovia* (*Nasonovia*) *ribisnigri* (Mosley, 1841)	+					
98	*Paczoskia longipes* (Tashev, 1964)	+					
99	*Phorodon* (*Phorodon*) *humuli* (Schrank, 1801)	+					
100	*Pterocomma populeum* (Kaltenbach, 1843)			+			
101	*Pterocomma rufipes* (Hartig, 1841)			+ *	+		
102	*Pterocomma tremulae* Börner, 1940			+ *			
103	! *Rhodobium porosum* (Sanderson, 1900)			+ *			
104	*Rhopalomyzus* (*Judenkoa*) *lonicerae* (Siebold, 1839)	+					
105	*Uroleucon* (*Uroleucon*) *sonchi* (Linnaeus, 1767)	+					
106	*Uroleucon* (*Uroleucon*) *telekiae* (Holman, 1965)	+					
107	! *Wahlgreniella nervata* (Gillette, 1908)			+			
Lachninae: Eulachnini							
108	*Cinara* (*Cinara*) *cuneomaculata* (Del Guercio, 1909)	+					
108	! *Cinara* (*Cinara*) *curvipes* (Patch, 1912)				+		
110	*Cinara* (*Cinara*) *piceae* (Panzer, 1800)		+				
111	*Cinara* (*Cinara*) *pilicornis* (Hartig, 1841)		+				
112	! *Essigella californica* (Essig, 1909)				+		
Total	112	60	51	31	32	11	6

## Data Availability

The original contributions presented in the study are included in the article and Supplementary Materials; further inquiries can be directed to the corresponding author.

## Supplementary Materials

The following supporting information can be downloaded at: https://www.mdpi.com/article/10.3390/insects17020196/s1, Table S1: Aphids collected in the Zabrze Botanic Garden, Poland in 2023, Table S2: Aphids collected in the Meise Botanic Garden, Belgium in 2023, Table S3: Aphids collected in the Meise Botanic Garden, Belgium in 2022, Table S4: Aphids collected in the Royal Botanic Garden Edinburgh, UK in 2023, Table S5: Aphids collected in the Royal Botanic Garden Benmore, UK in 2023, Table S6: Aphids collected in the Royal Botanic Garden Logan, UK in 2023. Table S7: Practical Protocol for Monitoring Aphids in Botanic Gardens.
