# Supplementary material for: Aphids (Hemiptera: Aphididae) in Living Collections of Selected European Botanic Gardens: Diversity, Biosecurity Challenges, and Sentinel Insights
Source: Insects. 2026 Feb 12;17(2):196. doi: 10.3390/insects17020196 (PMC12940903; doi:10.3390/insects17020196)
Supplement: Supplementary file 1 [file insects-17-00196-s001.zip › Table S7.pdf]

Table S7: Practical Protocol for Monitoring Aphids in Botanic Gardens

1. Survey Design and Timing

- **Frequency**: Intensive, systematic, full-garden surveys day over 2–3 consecutive days, conducted once per season (spring, summer, autumn), with additional supplementary monthly checks inspections in high-risk areas (nurseries, glasshouses, plant shops, quarantine areas). Intensive and monthly surveys should be carried out in the same major garden sections, ensuring consistency between visits.
- **Target periods**: Early spring (aphid colonization), late spring/early summer (population peak), and late summer (migration and secondary host use).
- **Duration**: Surveys should last at least 2–3 days to capture phenological variation and ensure repeat sampling across garden sections.

2. Standardised Sampling Methods

- **Plant selection strategy**

All accessible woody hosts, ornamental herbaceous plants, and representative native vegetation should be inspected. In nurseries, conservatories, and glasshouses all accessible plants must be checked regardless of origin or size. Native plants should be included because alien aphids may establish unexpected associations on local hosts.

- **Plant parts examined**

Inspect all plants' parts where aphids typically occur: young shoots and terminal growth, leaves, stems and petioles, buds and inflorescences.

- **Environmental conditions**

Record notable microclimatic differences (e.g., increased humidity in glasshouses, shaded vs. exposed areas), as they can influence aphid presence and colony persistence.

- **Collection method**

Aphids should be collected through careful visual inspection of suitable plant parts, supplemented, where appropriateby gently beating tree or shrub branches over a tray or white card to dislodge free-living species, particularly on conifers. Collect adult individuals into 70% ethanol by fine brush; record date, location, and host plant.

3. Host Plant Priorities

- **Curated collections**: Prioritize taxa with known aphid associations.
- **National or thematic collections**: Target unique holdings (e.g., national collections, endangered species).
- **Exotics under glass**: Inspect ornamental and tropical plants in nurseries and conservatories, as these are frequent entry points for alien species.
- **Native vegetation within or bordering the garden**: Important for detecting spill-over by alien aphids or previously undocumented host use.

4. Data Recording Standards

- **Metadata**: Host plant species (with accession number if available), garden section, aphid life stage (winged, wingless), parasitism, predation, presence of mutualistic ants, collection date, collector name.
- **Abundance estimate**: Scale (e.g., 1 = few individuals, 2 = scattered colonies, 3 = heavy infestation).
- **Damage symptoms**: Note presence/absence of leaf curl, chlorosis, defoliation, sooty mold.

5. Biosecurity Measures

- **Rapid response**: Notify horticultural staff and plant health officers upon detection of targeted species.
- **Isolation**: Move infested plants in nurseries to quarantine zones where possible.
- **Control methods**: Use garden-appropriate measures (water jetting, biological control, minimal chemical control where required).

6. Integration with Sentinel Networks

- **Identification**: Confirm species identity via keys or molecular barcoding if uncertain.
- **Reporting**: Upload verified records to national monitoring schemes and the International Plant Sentinel Network (IPSN).
- **Data sharing**: Encourage exchange of results between gardens to identify shared trends and potential invaders.
